# Unsafe Drinking Water Is Associated with Environmental Enteric Dysfunction and Poor Growth Outcomes in Young Children in Rural Southwestern Uganda

**DOI:** 10.4269/ajtmh.18-0143

**Published:** 2018-10-22

**Authors:** Jacqueline M. Lauer, Christopher P. Duggan, Lynne M. Ausman, Jeffrey K. Griffiths, Patrick Webb, Bernard Bashaasha, Edgar Agaba, Florence M. Turyashemererwa, Shibani Ghosh

**Affiliations:** 1 Gerald J. and Dorothy R. Friedman School of Nutrition Science and Policy, Tufts University, Boston, Massachusetts;; 2 United States Agency for International Development (USAID) Feed the Future Innovation Lab for Nutrition, Tufts University, Boston, Massachusetts;; 3 Division of Gastroenterology, Hepatology and Nutrition, Boston Children’s Hospital, Boston, Massachusetts;; 4 Department of Nutrition, Harvard T.H. Chan School of Public Health, Boston, Massachusetts;; 5 Department of Public Health and Community Medicine, Tufts University School of Medicine, Boston, Massachusetts;; 6 Tufts University Cummings School of Veterinary Medicine, Tufts University School of Engineering, Medford, Massachusetts;; 7 Department of Agribusiness and Natural Resource Economics, Makerere University, Kampala, Uganda;; 8 School of Food Technology and Nutrition, Makerere University, Kampala, Uganda

## Abstract

Environmental enteric dysfunction (EED), a subclinical disorder of the small intestine, and poor growth are associated with living in poor water, sanitation, and hygiene (WASH) conditions, but specific risk factors remain unclear. Nested within a birth cohort study, this study investigates relationships among water quality, EED, and growth in 385 children living in southwestern Uganda. Water quality was assessed using a portable water quality test when children were 6 months, and safe water was defined as lacking *Escherichia coli* contamination. Environmental enteric dysfunction was assessed using the lactulose:mannitol (L:M) test at 12–16 months. Anthropometry and covariate data were extracted from the cohort study, and associations were assessed using linear and logistic regression models. Less than half of the households (43.8%) had safe water, and safe versus unsafe water did not correlate with improved versus unimproved water source. In adjusted linear regression models, children from households with safe water had significantly lower log-transformed (ln) L:M ratios (β: −0.22, 95% confidence interval (CI): −0.44, −0.00) and significantly higher length-for-age (β: 0.29, 95% CI: 0.00, 0.58) and weight-for-age (β: 0.20, 95% CI: 0.05, 0.34) *Z*-scores at 12–16 months. Furthermore, in adjusted linear regression models, ln L:M ratios at 12–16 months significantly decreased with increasing length-for-age *Z*-scores at birth, 6 months, and 9 months (β: −0.05, 95% CI: −0.10, −0.004; β: −0.06, 95% CI: −0.11, −0.006; and β: −0.05, 95% CI: −0.09, −0.005, respectively). Overall, our data suggest that programs seeking to improve nutrition should address poor WASH conditions simultaneously, particularly related to household drinking water quality.

## Introduction

An estimated 155 million children less than 5 years of age are stunted, that is, have a length/height-for-age *Z*-score (LAZ/HAZ) of less than −2.^1^ Stunting is associated with an array of health and economic consequences, including a greater risk of infections in childhood, diminished cognitive development, poorer educational outcomes, and lower economic productivity and earnings in adulthood.^2^ However, despite the enormous global burden, mechanisms underlying stunting remain largely underexplored. That is, known interventions to resolve stunting implemented at 90% coverage would only avert 20% of the global burden, leaving most of the problem unaddressed.^3^ One of the domains of potential concern for stunting is poor environmental conditions (water, sanitation, and hygiene [WASH]) and associated intestinal health.

Some studies have demonstrated an association between poor WASH and poor growth outcomes,^4–7^ but the assumption that repeated symptomatic diarrheal infections are the main mechanism at work has not been supported. According to the 2008 Lancet Maternal and Child Nutrition Series, WASH interventions implemented at 99% coverage would reduce diarrhea incidence by 30%, which would reduce the prevalence of stunting by only 2.4% at 36 months of age.^8^ Furthermore, in a pooled analysis of nine studies, only 25% of stunting at 24 months was attributable to a high burden of diarrhea (≥ 5 episodes before 24 months).^9^

That diarrheal infection is not more strongly linked to stunting as an outcome has promoted the hypothesis that the impact of poor WASH on nutrition operates through environmental enteric dysfunction (EED).^10^ Environmental enteric dysfunction is a subclinical, inflammatory disorder of the small intestine characterized by altered gut morphology, reduced absorptive capacity, and impaired barrier function.^11,12^ It is postulated that EED develops throughout infancy as the result of chronic fecal–oral exposure to enteropathogens because of living in poor WASH conditions. However, to date, only a few studies have implicated WASH-related risk factors as being associated with EED, including unsafe child feces disposal,^13^ mouthing of soil (geophagy),^14^ and exposure to animals,^15^ whereas several studies have implicated exposure to specific enteropathogens, including *Giardia*,^16^ *Shigella*,^17^ and rotavirus.^18,19^ Furthermore, a study from Bangladesh found that children living in environmentally clean households had better intestinal health, characterized by lower lactulose:mannitol (L:M) ratios (−0.32 standard deviations [SDs], 95% CI: −0.72, 0.08), and higher HAZ (0.54 SDs, 95% CI: 0.06, 1.01) than children from contaminated households.^20^ However, despite these findings, the exact risk factors for EED remain speculative, inconsistent across studies, and in need of further study.

Although typically considered asymptomatic, EED is significant mainly because of its postulated association with poor growth outcomes, especially stunting, likely as the result of both malabsorption of nutrients and systemic immune activation.^21–26^ The primary objective of this study was to investigate the relationships among water quality, EED, and growth among children aged 12–16 months living in rural southwestern Uganda. We hypothesized that 1) children from households with unsafe drinking water would have higher levels of EED, measured using a L:M test, and 2) children with prior poor growth, particularly lower LAZ, would have higher levels of EED.

## Materials and methods

### Approvals.

The study was approved by the Tufts Health Sciences Institutional Review Board in Boston, MA; the Institutional Review Board at Harvard T.H. Chan School of Public Health, Boston, MA; the Makerere University Research Ethics Committee at the School of Public Health in Kampala, Uganda; and the Uganda National Council for Science and Technology in Kampala, Uganda. Before enrollment in the study, written consent was obtained from the child’s main caretaker.

### Study design.

This was a cross-sectional, observational study conducted as a sub-study to the Uganda Birth Cohort Study (UBCS) between July and August 2016 in seven subcounties (Bugangari, Buyanja, Bwizi, Kebisoni, Kibiito, Nyamweru, and Ruhiija) of rural southwestern Uganda. The UBCS was a prospective, observational study in 16 subcounties across northern and southwestern Uganda that enrolled ∼5,000 women and followed them up through pregnancy, birth, and through 9 months of the infant’s life.

### Sample size and eligibility.

The study reported here included a randomly selected sample of 385 children from the UBCS (i.e., 55 from each of the seven subcounties). Sample size was calculated with G*Power software (University of Düsseldorf, Düsseldorf, Germany) for a multiple regression with the following parameters: medium effect size (*ƒ*^2^ = 0.15),^27^ 0.95 power, 0.05 type 1 error probability, and seven predictors. Sample size was doubled to account for subcounty clustering and further increased by 25% to allow for potential challenges in conducting the L:M test (e.g., test failures due to urine leakage, contamination by stool, vomiting, and refusal to drink the solution).

Of the 4,951 women initially enrolled in the UBCS, 2,128 were from one of the seven subcounties selected for this study, and 2,015 births were successfully recorded. Children eligible for this substudy were between the ages of 12 and 16 months, capturing a period of elevated L:M ratios observed in previous studies,^28,29^ and they had complete UBCS visits up to 9 months. Based on these criteria, 562 children were eligible for selection into the study. We did not observe significant differences between eligible children and ineligible children from the same seven subcounties with respect to demographics, household characteristics, and growth outcomes. On the day of the L:M test, children were excluded from the study if they had had one or more episodes of diarrhea in the previous 2 weeks, were severely malnourished (mid-upper arm circumference < 11.5 cm), or had a serious illness.

### Lactulose:mannitol test.

Environmental enteric dysfunction in this study was measured using the L:M dual sugar absorption test. Although EED can only be diagnosed definitively through small intestinal biopsy,^30^ the L:M test is the most commonly used, noninvasive proxy marker.^31^ In the test, mannitol recovery rates indicate absorptive capacity, lactulose recovery rates indicate permeability, and higher L:M ratios indicate greater intestinal abnormality, or EED.^32^

Standardized doses, consisting of a 20 mL solution containing 5 grams of lactulose (Lactulose Solution; Mckesson, San Francisco, CA) and 1 gram of mannitol powder (D-mannitol powder; Sigma-Aldrich, St. Louis, MO) completely dissolved in sterile water, were prepared in the Food Science laboratory at Makerere University in Kampala and transferred to refrigerators located in local health facilities. After the consent process and an observed 1-hour fast, children were given a dose of solution using either a plastic cup or a disposable dropper. Each child was carefully monitored to ensure that none of the solution was spilled, spit out, or vomited. If any of these events occurred, the test was rescheduled for a different day.

After children successfully consumed the L:M solution, urine was collected over a minimum of a 4-hour period using sterile adhesive urine collection bags (Thermo Fisher Scientific, Waltham, MA), which were replaced after each urination episode. Collected urine was consolidated in a plastic container with thimerosal (Sigma-Aldrich) added to the container to prevent bacterial growth. Drinking water was provided and allowed ad libitum throughout the test, and breastfeeding and/or a small meal was allowed at the 3-hour mark. At the 4-hour mark, children were offered a juice drink to encourage a final urination episode, which marked the end of the test.

Total urine volume was measured to the nearest 1.0 mL using a graduated cylinder and the urine was aliquoted into plastic cryovials. The samples were transported on ice in plastic cooler boxes to the local health facility where they were stored at −20°C. On completion of the study, samples were stored at −80°C in Kampala before being transported on dry ice to the laboratory at Baylor College of Medicine for analysis. Concentrations of lactulose and mannitol were analyzed using high-performance liquid chromatography using previously described methods.^33,34^

### Anthropometry and covariates.

With the exception of the L:M data, data required for analysis were extracted from the UBCS main dataset. Data were collected by trained research assistants at 3-month intervals using electronic tablets from pregnancy until the infant turned 9 months of age. Information included household characteristics, WASH, diet, health, food security, gender and decision-making, agricultural production, and anthropometry. Covariates were obtained from the 6-month time-point and anthropometry measurements were obtained at birth, 6 months, and 9 months as well as at the time of the L:M test.

Length was measured in triplicate to the nearest 0.1 cm using a portable height board (ShorrBoard^®^ infant/child/adult portable height-length measuring board; Weigh and Measure, LLC, Olney, MD) and weight was measured in triplicate to the nearest and 0.1 kg using an electronic scale (Seca, Hanover, MD). Triplicate measurements were averaged to provide one measurement of length and weight, per participant per visit. Hemoglobin was measured at 6 months of age using a portable hemoglobinometer (HemoCue 301; HemoCue America, Brea, CA).

### Water quality.

Water quality was assessed in the UBCS using a compartment bag test (CBT kit; Aquagenx, Chapel Hill, NC)^35^ at the 6-month time-point. The compartment bag test (CBT) is a portable water quality test kit designed to detect and quantify *Escherichia coli* bacteria. For the test, participants were asked to provide a glass of water from their primary drinking water storage container and a 100 mL sample was mixed with an *E. coli* chromogenic growth medium. The sample was then poured into a plastic bag with five compartments of varying volumes, sealed, and incubated for a period of 48 hours. Risk categories were determined by noting which, if any, compartments changed from yellow to green/blue and matching that to a most probable number table based on the World Health Organization (WHO) guidelines. Health risk categories are safe (< 1 CFU [colony-forming unit]/100 mL), intermediate risk (1–10 CFU/100 mL), high risk (> 10–100 CFU/100 mL), and very high risk/unsafe (> 100 CFU/100 mL).^36^

### Statistical methods.

All analyses were carried out using STATA 15 software (Stata Corps, College Station, TX). The primary outcomes of interest were the log-transformed (ln) L:M ratio, measured at 12–16 months, and LAZ at birth, 6 months, 9 months, and the time of the L:M test. Secondary outcomes included percent lactulose excretion (%LE), percent mannitol excretion (%ME), and the lactulose mannitol excretion ratio (LMER) in addition to weight-for-age (WAZ) and weight-for-length (WLZ) *Z*-scores.

Lactulose:mannitol ratios were calculated using the fractional excretion of each of the two sugars. Because of the right-skewed nature of its distribution, L:M ratios were natural ln before all regression analyses. Percent lactulose excretion and %ME were calculated by first multiplying the concentration of sugar (mg/mL) by the total urine volume and then dividing that amount by the initial dose of each sugar. The LMER was calculated by taking the ratio of %LE to %ME.

Growth outcomes, including LAZ, WAZ, and WLZ, were calculated using the WHO Multicenter Growth Reference Study growth standards.^37^ Dichotomous variables, stunting (LAZ < −2), underweight (WAZ < −2), and wasting (WLZ < −2) were also created. All extreme outliers (−6 > WAZ > 5, −6 > LAZ > 6, and −5 > WLZ > 5) were set to missing, as per WHO recommendations. Anemia was defined as hemoglobin < 11 g/dL.

A dichotomous (safe versus unsafe) water variable was created, with safe water defined as no *E. coli* detected in the CBT and unsafe water defined as any *E. coli* detected. Improved water sources were piped water, a public tap, a tube well/borehole, a protected well/spring, and rain water. Unimproved water sources were an unprotected well/spring, surface water, and other. An asset score was created based on the simple sum of households’ ownership of the following four items: telephone, bicycle, radio, and motorcycle.

Associations among water quality, L:M results, and growth were assessed using unadjusted and adjusted linear and logistic regression models. For all regression models, subcounty clustering was controlled for using generalized estimating equations. For all adjusted models, covariates were selected based on bivariate analyses with the primary outcomes (i.e., ln L:M ratio or LAZ) and a *P*-value cut point of 0.20. Variance inflation factor was used to verify a lack of collinearity among predictors. Associations were considered significant in the case of *P*-value < 0.05.

## Results

### Study population.

Background characteristics of the 385 participating children and their households are presented in Table 1. Half of the children were female and mean age at enrollment into the sub-study was ∼15 months. As expected, mean LAZ, WLZ, and WAZ declined over time from birth until the L:M test. At the time of the L:M test, 35.1% of the participants were stunted, 8.8% were underweight, and 2.1% were wasted.

**Table 1:** Characteristics of 385 Ugandan children and their households

Characteristic	Mean ± SD or *n* (%)
Child characteristics	
Female	195 (50.7)
Age, months	14.8 ± 1.1
Anthropometry at birth	
Length-for-age *Z*-score	−0.93 ± 1.54
Weight-for-length *Z*-score	0.57 ± 1.54
Weight-for-age *Z*-score	−0.17 ± 0.96
Anthropometry at 6 months of age	
Length-for-age *Z*-score	−0.98 ± 1.51
Weight-for-length *Z*-score	0.63 ± 1.41
Weight-for-age *Z*-score	−0.25 ± 1.19
Anthropometry at 9 months of age	
Length-for-age *Z*-score	−1.21 ± 1.46
Weight-for-length *Z*-score	0.42 ± 1.35
Weight-for-age *Z*-score	−0.41 ± 1.21
Anthropometry at L:M test	
Length-for-age *Z*-score	−1.55 ± 1.14
Weight-for-length *Z*-score	0.24 ± 1.10
Weight-for-age *Z*-score	−0.58 ± 1.04
Hemoglobin at 6 months of age, g/dL	11.2 ± 1.2
L:M ratio	0.34 ± 0.27
Urinary lactulose, % dose excreted	0.32 ± 0.28
Urinary mannitol, % dose excreted	5.32 ± 3.48
LMER	0.07 ± 0.05
Household characteristics	
Individuals in household	5.7 ± 2.4
Female household head	16 (4.2)
Caregiver education years	5.9 ± 3.0
Earth floor	334 (86.8)
Electricity, grid/solar	61 (15.8)
Unimproved pit latrine	368 (95.6)
Water quantity, jerrycans per day	2.4 ± 1.3
Boil water	274 (71.2)
Water quality (*n* = 377)	
Safe	165 (43.8)
Intermediate risk	51 (13.5)
High risk	46 (12.2)
Very high risk	115 (30.5)

L:M = lactulose:mannitol; LMER = L:M excretion ratio.

On average, households had approximately six members, and most dwellings had an earth floor, an unimproved pit latrine, and no electricity. Table 2 shows the percentage of households with safe versus unsafe water, disaggregated by main water source. Among 377 sampled households, 43.8% had safe water and 56.2% had unsafe water. We observed no correlation between having an improved water source and having safe drinking water. Among the 210 households with an improved water source, 44.3% had safe water and 55.7% had unsafe water. Similarly, among the 167 households with an unimproved water source, 43.3% had safe water and 56.7% had unsafe water (chi-square *P*-value = 0.82). Furthermore, we observed no significant associations between water source and either EED risk or stunting risk.

**Table 2:** Comparison of water quality (safe vs. unsafe)* by main water source among 377 households in southwestern Uganda

Main water source	Total	Safe, *n* (%)	Unsafe, *n* (%)
Piped	8	4 (50.0)	4 (50.0)
Public tap	45	26 (57.8)	19 (42.2)
Tube well/borehole	57	17 (29.8)	40 (70.2)
Protected well/spring	85	35 (41.2)	50 (58.8)
Unprotected well/spring	110	54 (49.1)	56 (50.9)
Rain water	15	11 (73.3)	4 (26.7)
Surface water	54	17 (31.5)	37 (68.5)
Other	3	1 (33.3)	2 (66.7)
Total	377	165 (43.8)	212 (56.2)

According to the World Health Organization, improved drinking water sources are piped water, public taps, tube wells/boreholes, protected wells/springs, and rainwater. Unimproved sources are unprotected wells/springs and surface water.

* Safe water is defined as the lack of the presence of *Escherichia coli* contamination according to the results of a compartment bag test. Unsafe water is defined as any *E. coli* contamination detected.

### Lactulose:mannitol results.

The arithmetic mean ± SD L:M ratio for participants was 0.34 ± 0.27. Table 3 shows the association between water quality and L:M test results in unadjusted and adjusted linear regression models. In adjusted linear regression models, L:M ratios were significantly lower in children from households with safe versus unsafe water (β: −0.22, 95% CI: −0.44, −0.00) as was the %LE (β: −0.08, 95% CI: −0.14, −0.01). No significant difference in %ME was observed between households with safe versus unsafe water.

**Table 3:** Association between water quality (safe vs. unsafe)† and L:M test results in unadjusted and adjusted linear regression models‡

	Unadjusted linear regression model	Adjusted linear regression model
Ln L:M ratio	−0.23 (−0.47, 0.00)*	−0.22 (−0.44, 0.00)*
Urinary lactulose, % dose excreted	−0.09 (−0.16, −0.02)*	−0.08 (−0.14, −0.01)*
Urinary mannitol, % dose excreted	−0.26 (−1.13, 0.60)	−0.09 (−1.05, 0.87)
LMER	−0.02 (−0.04. 0.003)	−0.02 (−0.04, 0.01)

L:M = lactulose:mannitol; LMER = L:M excretion ratio. Cells present β coefficient and 95% confidence interval, * *P*-value < 0.05.

† Safe water is defined as the lack of the presence of *Escherichia coli* contamination according to the results of a compartment bag test. Unsafe water is defined as any *E. coli* contamination detected.

‡ Unadjusted and adjusted regression models adjusted for subcounty clustering. Adjusted regression model controls for gender of child, gender of household head, mother’s height, caregiver education level, family size, and asset score.

### Association between water quality and growth outcomes.

Table 4 shows the association between water quality and growth outcomes (LAZ, WAZ, and WLZ) using unadjusted and adjusted linear regression models. In addition to better intestinal health, children from households with safe water had significantly better overall LAZ and WAZ, but not WLZ. In adjusted linear regression models, safe water was significantly associated with better LAZ at birth (β: 0.57, 95% CI: 0.10, 1.04) and at the time of the L:M test (β: 0.29, 95% CI: 0.00, 0.58). Furthermore, in adjusted linear regression models, safe water was significantly associated with better WAZ at 6 months (β: 0.23, 95% CI: 0.06, 0.41) and at the time of the L:M test (β: 0.20, 95% CI: 0.05, 0.34).

**Table 4:** Association between water quality (safe vs. unsafe)† and growth outcomes (LAZ, WAZ, and WLZ) at birth, 6 months, 9 months, and the time of the L:M test in unadjusted and adjusted linear regression models‡

Outcome	Unadjusted linear regression model	Adjusted linear regression model
Growth at birth		
LAZ	0.65 (0.06, 1.24)*	0.57 (0.10, 1.04)*
WAZ	0.18 (−0.06, 0.43)	0.15 (−0.12, 0.42)
WLZ	−0.38 (−1.04, 0.28)	−0.38 (−1.02, 0.27)
Growth at 6 months		
LAZ	0.40 (−0.27, 1.08)	0.16 (−0.24, 0.56)
WAZ	0.35 (0.18, 0.52)*	0.23 (0.06, 0.41)*
WLZ	−0.02 (−0.64, 0.60)	0.13 (−0.32, 0.58)
Growth at 9 months		
LAZ	0.25 (−0.40, 0.89)	0.10 (−0.28, 0.48)
WAZ	0.35 (0.11, 0.60)*	0.23 (−0.03, 0.49)
WLZ	0.18 (−0.33, 0.69)	0.16 (−0.31, 0.63)
Growth at L:M test (12–16 months)		
LAZ	0.39 (0.13, 0.65)*	0.29 (0.00, 0.58)*
WAZ	0.29 (0.16, 0.43)*	0.20 (0.05, 0.34)*
WLZ	0.08 (−0.24, 0.40)	0.14 (−0.17, 0.44)

L:M = lactulose:mannitol; LAZ = length-for-age *Z*-score; WAZ = weight-for-age *Z*-score; WLZ = weight-for-length *Z*-score. Cells present β coefficient and 95% confidence interval, * *P*-value < 0.05.

† Safe water is defined as the lack of the presence of *Escherichia coli* contamination according to the results of a compartment bag test. Unsafe water is defined as any *E. coli* contamination detected.

‡ Unadjusted and adjusted regression models adjusted for subcounty clustering. Adjusted regression models control for gender of child, gender of household head, mother’s height, caregiver education level, family size, and asset score. Adjusted regression model for 6 months, 9 months, and the L:M test time-point controls for LAZ, WAZ, and WLZ, respectively, at birth.

Finally, in adjusted logistic regression models, the odds of being stunted were 1.68 (95% CI: 1.22, 2.32), 1.70 (95% CI: 1.21, 2.37), and 1.38 (95% CI: 0.88, 2.18) times higher for those from households with unsafe water than for those with safe water, at birth, 6 months, and the time of the L:M test, respectively (Supplemental Table 1). The odds of being anemic at 6 months were 1.63 times higher (95% CI: 0.88, 3.04) for those from households with unsafe water than for those with safe water.

### Association between intestinal health and past LAZ.

Higher L:M ratios were observed in children who were stunted than in those not stunted at birth (0.10-point difference, 95% CI: 0.04, 0.16), 6 months (0.10-point difference, 95% CI: 0.03, 0.16), and 9 months (0.07-point difference, 95% CI: 0.004, 0.13). Table 5 shows the association between past LAZ and L:M results in unadjusted and adjusted linear regression models. In adjusted linear regression models, higher LAZ at birth, 6 months, and 9 months were significantly associated with lower ln L:M ratios at 12–16 months (β: −0.05, 95% CI: −0.10, −0.004; β: −0.06, 95% CI: −0.11, −0.006; and β: −0.05, 95% CI: −0.09, −0.005, respectively).

**Table 5:** Association between prior length-for-age *Z*-scores, at birth, 6 months, and 9 months, and L:M test results in unadjusted and adjusted linear regression models†

	Unadjusted linear regression model			Adjusted linear regression model		
Outcome	LAZ at birth	LAZ at 6 months	LAZ at 9 months	LAZ at birth	LAZ at 6 months	LAZ at 9 months
Ln L:M ratio	−0.06 (−0.13, 0.00)*	−0.07 (−0.17, 0.03)	−0.05 (−0.12, 0.03)	−0.05 (−0.10, −0.004)*	−0.06 (−0.11, −0.006)*	−0.05 (−0.09, −0.005)*
Urinary lactulose, % dose excreted	−0.009 (−0.03, 0.02)	−0.02 (−0.06, −0.02)	−0.02 (−0.05, 0.009)	−0.0003 (−0.02, 0.02)	−0.01 (−0.04, 0.02)	−0.02 (−0.05, 0005)
Urinary mannitol, % dose excreted	0.17 (−0.11, 0.45)	0.03 (−0.29, 0.36)	−0.01 (−0.33, 0.31)	0.26 (0.02, 0.50)*	0.09 (−0.21, 0.39)	0.02 (−0.27, 0.30)
LMER	−0.004 (−0.009, 0.00)*	−0.006 (−0.01, −0.002)	−0.004 (−0.01, 0.002)	−0.004 (−0.008, 0.00)*	−0.005 (−0.01, 0.0005)	−0.004 (−0.009, 0.001)

L:M = lactulose:mannitol; LMER = L:M excretion ratio; LAZ = length-for-age *Z*-score. Cells present β coefficient and 95% confidence interval, * *P*-value < 0.05.

† Unadjusted and adjusted regression models were adjusted for subcounty clustering. Adjusted regression models control for gender of child, age of child, gender of household head, caregiver education level, asset score, and safe water. Adjusted regression model for LAZ at 6 months and 9 months controls for LAZ at birth.

## Discussion

In this study of 385 children living in rural southwestern Uganda, we found that those from households with safe drinking water, assessed using a CBT at the 6-month time-point, had significantly lower ln L:M ratios and %LE at 12–16 months. In addition, at 12–16, months, children from households with safe water had 0.29 *Z*-score higher LAZ and 0.20 *Z*-score higher WAZ on average. Finally, we found that lower LAZ at birth, 6 months, and 9 months were significantly associated with higher mean ln L:M ratios at 12–16 months. Overall, these results add to the growing body of literature connecting poor WASH conditions, EED, and poor growth. Specifically, our results indicate that contaminated household drinking water may be an important contributing factor to the high burden of both EED and stunting in southwestern Uganda and in other low- and middle-income countries with poor WASH conditions.

To our knowledge, this is one of the first studies to use an objective measure of drinking water quality to link both EED and poor child growth with a specific water-borne enteric pathogen, in this case *E. coli.* Conventionally, water quality is assessed using a sole indicator of “improved drinking water source” versus “unimproved drinking water source.”^36^ However, had this definition of water quality been used, we would have observed no association between water quality and either EED risk or stunting risk in this study. Our finding that safe versus unsafe water did not correlate with improved versus unimproved water source is consistent with findings from rural Peru, where the authors used the same CBT and determined that improved water sources were not associated with decreased contamination risk.^38^ This lack of correlation is speculatively the result of other contamination sources, including poor management of water sources, poor storage practices, and water-related behaviors that can increase the risk of post-collection contamination. Together, these findings demonstrate a need for more objective WASH indicators that adequately assess risk of exposure to pathogens.

Furthermore, our study adds to the growing body of literature that supports a link between poor WASH conditions and the development of EED in young children. In a study by Lin et al.^20^ of 119 rural Bangladeshi children ≤ 48 months of age, the authors assessed the relationship between fecal environmental contamination and EED. They found that children living in environmentally “clean” households, defined using objective indicators of water quality and sanitary and handwashing infrastructure, had better intestinal health, characterized by lower L:M ratios (−0.32 SDs, 95% CI: −0.72, 0.08), than children from “contaminated” households. In addition, in a prospective cohort study of 216 children < 5 years of age also in rural Bangladesh, George et al.^14^ observed an association between geophagy (i.e., consumption of soil, dirt, or mud) and EED as well as between animal exposure and caregiver hygiene and EED, measured using four fecal markers: alpha-1-antitrypsin, myeloperoxidase, and neopterin (all three combined to form an EED disease activity score) and calprotectin. Children with caregiver-reported geophagy had significantly higher EED scores (0.72-point difference, 95% CI: 0.01, 1.42) and calprotectin concentrations (237.38 µg/g, 95% CI: 12.77, 462.00). Furthermore, children with an animal corral in their sleeping room had significantly higher EED scores (1.0-point difference, 95% CI: 0.13, 1.88) and children of caregivers with visibly soiled hands had significantly higher fecal calprotectin concentrations (384.1 µg/g, 95% CI: 152.37, 615.83).^15^

This study has several limitations, and it also points to several areas in need of further research. First, both the L:M test and CBT water quality test have inherent disadvantages. The L:M test, although still the most commonly used measure of EED in the field, suffers from significant variability in methods related to aspects such as fasting time, dosing amount, urine collection time, and laboratory analysis. Furthermore, there is debate regarding both the L:M test’s ability to adequately assess EED and its correlation with poor growth.^31^ The CBT water quality test, although a convenient, inexpensive method of measuring water quality in the field, provides a statistical “most likely mean” measure of CFUs per 100 mL and approximate risk categories rather than a precise measure of contamination.

Although certain data elements in this study were collected prospectively, other elements, notably the L:M test and the CBT, were measured at a single time point, restricting our capacity to establish causality. In particular, measuring water quality at only one point in time fails to capture the often-extreme temporal variability in this indicator, which is problematic as EED is likely the result of cumulative contact with enteropathogens over time rather than one-time exposure. In addition, anthropometry data were not collected beyond the point of the L:M test, and therefore we can only conclude that L:M results were associated with past growth rather than future growth. Finally, as we tested numerous outcomes in this study, we must acknowledge the possible effect of multiple comparisons.

Despite the fact that *E. coli* is the preferred indicator of fecal contamination, the authors acknowledge that measuring *E. coli* alone is an imperfect proxy for water contamination. In addition to *E. coli*, it would be useful to look at the association between other pathogens, including *Cryptosporidium*, *Shigella*, *Salmonella*, and viruses, and their relationship with EED. Furthermore, in addition to EED and iron status, it would be useful to examine the role of EED in other micronutrient deficiencies, including addition to zinc, vitamin A, folate, and vitamin B12. Finally, given the significant association between household water quality and stunting at birth, it is worth exploring whether maternal EED is linked to certain negative birth outcomes, such as reduced in utero growth.

Moving forward, a randomized controlled trial nested within a prospective birth cohort study that offers improvements in household water quality would be a valuable next step to provide additional causal support of our hypothesis. From a programmatic perspective, WASH interventions should focus on preventing EED by reducing children’s fecal–oral exposure to enteropathogens. This should include an emphasis on improving water quality in settings where water contamination is prevalent and likely a predominate underlying cause of EED.

## Supplementary Material

Supplemental figure
