# Supplementary material for: Unsafe Drinking Water Is Associated with Environmental Enteric Dysfunction and Poor Growth Outcomes in Young Children in Rural Southwestern Uganda
Source: Am J Trop Med Hyg. 2018 Oct 22;99(6):1606–12. doi: 10.4269/ajtmh.18-0143 (PMC6283503; doi:10.4269/ajtmh.18-0143)
Supplement: Supplementary file 1 [file tpmd180143.SD1.pdf]

Appendix Table A1: Relationship between municipality TB rate and the presence of correctional facilities

	Specification 1					Specification 2					Specification 3				
Variable	Coef	Std. Err	p-value	Lower CI	Upper CI	Coef	Std. Err	p-value	Lower CI	Upper CI	Coef	Std. Err	p-value	Lower CI	Upper CI
At least one corr. Facil	0.299	0.096	0.002	0.110	0.488	0.244	0.091	0.008	0.065	0.423					
One corr. facility											0.241	0.092	0.009	0.062	0.421
Two corr. facilities											0.422	0.173	0.015	0.082	0.762
Three+ corr. facilities											0.627	0.148	0.000	0.337	0.916
Demographic controls															
Municipality population	0.000	0.000	0.083	0.000	0.000	0.000	0.000	0.000	0.000	0.000	0.000	0.000	0.016	0.000	0.000
Population density	0.000	0.000	0.530	0.000	0.001	0.000	0.001	0.919	-0.001	0.001	0.000	0.000	0.300	0.000	0.001
% Black African residents	-0.001	0.003	0.741	-0.007	0.005	-0.001	0.003	0.749	-0.006	0.004	0.000	0.003	0.937	-0.006	0.006
Males per 100 females	0.001	0.009	0.888	-0.017	0.019	0.002	0.008	0.769	-0.013	0.018	-0.002	0.008	0.804	-0.019	0.014
Socioeconomic controls															
Unemployment rate	-0.001	0.008	0.900	-0.017	0.015	-0.008	0.007	0.268	-0.021	0.006	-0.001	0.008	0.907	-0.017	0.015
No education	0.002	0.011	0.834	-0.020	0.024	0.014	0.011	0.203	-0.007	0.035	0.006	0.011	0.598	-0.016	0.028
Female headed household	-0.006	0.005	0.221	-0.016	0.004	-0.009	0.005	0.053	-0.018	0.000	-0.010	0.005	0.050	-0.020	0.000
Formal dwelling	-0.009	0.007	0.188	-0.022	0.004	-0.010	0.006	0.084	-0.021	0.001	-0.011	0.006	0.079	-0.023	0.001
Flush toilet	0.013	0.005	0.007	0.003	0.022	0.009	0.004	0.022	0.001	0.017	0.010	0.005	0.029	0.001	0.020
Garbage removal	-0.002	0.006	0.721	-0.013	0.009	-0.002	0.005	0.629	-0.012	0.007	0.001	0.005	0.896	-0.010	0.011
Piped water	-0.003	0.006	0.605	-0.015	0.009	-0.004	0.006	0.464	-0.015	0.007	-0.003	0.006	0.651	-0.015	0.009
Electricity	-0.004	0.008	0.605	-0.020	0.012	0.005	0.007	0.516	-0.009	0.019	-0.003	0.008	0.716	-0.019	0.013
Health system controls															
Under 5 mortality	0.028	0.043	0.507	-0.055	0.112	0.051	0.040	0.197	-0.027	0.128	0.036	0.042	0.393	-0.047	0.118
Immunization rate	-0.012	0.007	0.103	-0.026	0.002	-0.010	0.007	0.112	-0.023	0.002	-0.013	0.007	0.085	-0.028	0.002
MMR immunization rate	-0.001	0.001	0.386	-0.003	0.001	-0.002	0.001	0.030	-0.004	0.000	-0.001	0.001	0.633	-0.003	0.002
Condom distribution rate	-0.006	0.008	0.401	-0.022	0.009	0.000	0.007	0.964	-0.013	0.013	-0.006	0.008	0.448	-0.021	0.009
Smear conversion rate	0.017	0.013	0.180	-0.008	0.042	0.008	0.012	0.469	-0.014	0.031	0.015	0.013	0.236	-0.010	0.040
TB cure rate	0.015	0.012	0.195	-0.008	0.038	0.004	0.010	0.693	-0.016	0.024	0.010	0.012	0.386	-0.013	0.033
TB default rate	0.019	0.038	0.618	-0.056	0.094	-0.025	0.038	0.503	-0.099	0.049	-0.005	0.039	0.902	-0.082	0.072
HIV testing rate	0.009	0.017	0.576	-0.024	0.043	0.046	0.018	0.011	0.010	0.081	0.020	0.018	0.281	-0.016	0.056
Antenatal HIV+ test rate	-0.029	0.013	0.024	-0.054	-0.004	-0.017	0.011	0.117	-0.039	0.004	-0.029	0.014	0.030	-0.056	-0.003
Antenatal ART take-up	0.001	0.005	0.905	-0.008	0.009	-0.001	0.004	0.763	-0.010	0.007	0.001	0.005	0.818	-0.008	0.010
Govt. hlth. exp. uninsured	0.000	0.000	0.046	0.000	0.001	0.000	0.000	0.006	0.000	0.001	0.001	0.000	0.010	0.000	0.001
Spending on primary care	0.001	0.007	0.833	-0.011	0.014	0.006	0.006	0.289	-0.005	0.018	0.001	0.006	0.848	-0.011	0.013
Total number of tests						0.000	0.000	0.000	0.000	0.000					
Constant	-6.542	2.270	0.004	-10.992	-2.092	-9.398	2.373	0.000	-14.049	-4.748	-6.638	2.262	0.003	-11.071	-2.204
